# The evolution of medical students’ preparedness for clinical practice during the transition of graduation: a longitudinal study from the undergraduate to postgraduate periods

**DOI:** 10.1186/s12909-021-02679-8

**Published:** 2021-05-06

**Authors:** Chung-Hsien Chaou, Shiuan-Ruey Yu, Yu-Che Chang, Shou-De Ma, Hsu-Min Tseng, Ming-Ju Hsieh, Ji-Tseng Fang

**Affiliations:** 1 Chang-Gung Medical Education Research Centre, Chang Gung Memorial Hospital, Taoyuan City, Taiwan; 2 Department of Emergency Medicine, Chang Gung Memorial Hospital, Linkou, Taoyuan, Taiwan; 3 Chang Gung University College of Medicine, Taoyuan, Taiwan; 4 Department of Health Care Management, Chang Gung University, Taoyuan, Taiwan; 5 Department of Thoracic Surgery, Chang Gung Memorial Hospital, Linkou, Taiwan; 6 Department of Nephrology, Chang Gung Memorial Hospital, Linkou, Taoyuan, Taiwan

**Keywords:** Preparedness for practice, Undergraduate medical education, Professional identity, Longitudinal study, Questionnaire study, Transition, Clinical placement

## Abstract

**Background:**

Graduating from medical school and beginning independent practice appears to be a major transition for medical students across the world. It is often reported that medical graduates are underprepared for independent practice. Most previous studies on undergraduates’ preparedness are cross-sectional. This study aimed to characterize the development and trend of medical students’ preparedness and its association with other objective and subjective indicators from the undergraduate to postgraduate periods.

**Methods:**

This was a prospective cohort study. The participants were recruited and followed from two years before graduation to the postgraduate period. The preparedness for independent practice, professional identity, and teamwork experience were biannually measured using previously validated questionnaires. The participants’ basic demographic information, clinical learning marks from the last two years, and national board exam scores were also collected.

**Results:**

A total of 85 participants completed 403 measurements in the 5 sequential surveys. The mean age at recruitment was 23.6, and 58 % of participants were male. The overall total preparedness score gradually increased from 157.3 (SD=21.2) at the first measurement to 175.5 (SD=25.6) at the fifth measurement. The serial individual preparedness scores revealed both temporal differences within the same learner and individual differences across learners. Despite the variations, a clear, steady increase in the overall average score was observed. Participants were least prepared in the domain of patient management at first, but the score increased in the subsequent measurements. The participants with better final preparedness had better professional identity (*p*<0.01), better teamwork experience (*p* < 0.01), and higher average clinical rotation marks (*p*<0.05).

**Conclusions:**

The preparedness for practice of medical students from the undergraduate to postgraduate periods is associated with their professional identity, teamwork experience, and objective clinical rotation endpoint. Although preparedness generally increases over time, educators must understand that there are temporal fluctuations and individual differences in learners’ preparedness.

## Background

Medical education is a long and continuous process in which a medical student gradually transforms into a doctor with professional competencies. Depending on the learning methods, content, and setting, the process is often divided into several different stages, such as basic medicine education, clinical placements, residency, and faculty development [1–5]. Due to the relatively homogenous learning environment in each stage, learners are able to master the knowledge, skills, and attitudes that should be learned in that stage in a relatively routine way. However, during the transition from one stage to another, students may experience major changes [6].

Regardless of the country or curriculum system, one of the most substantial transitions in the process of medical education is the period before and after graduation [1, 7, 8]. Normally, after receiving basic medical training, medical students undertake a period of rotatory clinical observership before graduation. This period is called by different names, such as clerkship, (undergraduate) internship, or clinical placement [2, 3, 9]. Within this period, students’ clinical learning is directed by clinical educators, who are responsible for facilitating the acquisition of profession-specific skills while students are in the field [10, 11]. Then, students graduate and pass the board examination, after which they begin independent practice under supervision in the hospital. This postgraduate stage could be a (postgraduate) internship, postgraduate year (PGY) rotation, or residency, depending on the system [1, 2, 7].

During the transition of graduation, the trainees experience several major changes. The first is a transformation of identity: students become physicians through national examinations. For those who are not well prepared, it may seem that they are forced to become doctors overnight. Second, instead of paying tuition, with the goal of learning being the first priority, postgraduate learners are being paid, and in addition to learning, they now have competing duties, such as taking care of patients. In terms of the learning environment, preclinical education comprises mostly discipline-oriented classroom learning and problem-based discussions. In the hospital, training is usually case-based, involving hands-on learning with real patients. This clinical education involves learning clinical and professional skills and provides learners with the opportunity to actively incorporate theoretical knowledge into clinical practice [12]. Finally, the competencies needed may be different. Preclinical learning focuses on single-professional practice, whereas clinical education is generally team-based, with the learning of multispecialty or multi-professional competencies that help learners understand healthcare delivery complexities.

The major changes occurring during the transition from the undergraduate to postgraduate periods pose challenges to medical students worldwide [6, 13]. Although one of the major aims of medical schools is to prepare undergraduates for their subsequent postgraduate hospital practice in all competency dimensions, it is often reported that medical students are underprepared for independent practice [14, 15]. Monrouxe and her colleagues, in their national qualitative study, reported that the August transition period was the time of greatest stress for new graduates, with them feeling unprepared, especially for their perceived change in responsibility, workload, and multitasking [8, 15]. It has even been reported that underprepared new graduates may negatively impact the quality of care at teaching hospitals during the transition period [16, 17].

Preparedness refers to leaners’ reported sense of being prepared for a number of areas of practice. It implies that the learners themselves are aware of their capabilities and are confident in their ability to safely begin work [18]. Preparedness for practice is thought to be associated with increased feelings of self-efficacy and the acquisition of “generic skills’’, including problem-solving, critical thinking, and communication [19, 20]. Various measurements have been proposed for the preparedness of different competencies, and the results allow clinical educators to understand how ready, overall or in different domains of competencies, their medical students are to be doctors[18, 21]. It also enables the comparison of educational outcomes across the curriculum, especially for practical skills. Various results regarding preparedness for clinical practice have been reported across countries [20, 22–25]. However, most of these studies are cross-sectional, and the trend of preparedness during the transition of graduation has not been followed and studied. Knowledge of this evolutionary change may help clinical educators better prepare their learners to cope with this transition and change. This longitudinal study aims to understand the development and trend of medical students’ preparedness and its association with other objective and subjective indicators from the undergraduate to postgraduate periods.

## Methods

### Study setting

This was a prospective cohort study. The participants were recruited and followed from 2 years before graduation to the postgraduate period with repeated questionnaire surveys. During the first year of the study, they all completed their clinical rotation observership at Chang-Gung Memorial Hospital, Linkou branch. This is a tertiary medical center and the largest teaching hospital in Taiwan. In the second year, these students were assigned to four large teaching hospitals in Taiwan at their will to receive another year of clinical rotation. During this last year before graduation, they are provided with more hands-on learning opportunities and share more responsibility for patient care under supervision. At the end of the second year of study, they graduated and then took the national medical board exam. In the third year, they entered their PGY1 program through a national matching process at different teaching hospitals across Taiwan. The PGY period is a mandatory postgraduate rotatory program for all medical graduates before entering residency in any specialty.

### Participants and data collection

The participants were medical students from Chang-Gung University who graduated in 2019, regardless of age. Recruitment was performed in September 2017 via email and posters. Because of the long study period, all participants were given a one-hour recruitment orientation before providing written consent and were followed until January 2020. The basic demographic data collected included age, sex, hometown location, self-reported previous academic performance, and site of clinical learning. Our participants were asked to fill out previously validated online questionnaires related to their preparedness for independent practice, professional identity, and team collaboration biannually. Specifically, the time points for measurement were 18, 12, 6, and 1 month before graduation and 5 months after graduation. Each measurement was performed within a one-month period. We used SurveyMonkey as the online interface for the questionnaire survey. Objective learning endpoints, including clinical learning marks from the last two years and board exam scores, were also collected if the participants agreed to provide them. The marks in clinical rotations are predefined structured assessment results from each specialty, usually consisting of marks from clinical teachers (faculty), marks from residents on the same team, formative assessment results, meeting attendance, and written test results. The board exam score collected was from the last stage, a knowledge-based multiple choice question (MCQ) test for licensing qualification.

### Ethics approval and consent to participate

This study was approved by the Chang-Gung Institutional Review Board (IRB No. 201601758B0, 201701981B0). Consents were obtained from every participant.

### Instruments

Preparedness for independent clinical practice was measured by the Chinese version of the Preparedness for Hospital Practice Questionnaire (PHPQ). The original English version was developed by Hill et al.[26] and consisted of 41 items in eight subscale domains: self-directed learning (SDL), holistic care (HC), prevention (PV), science (SC), management (MG), collaboration (CL), confidence (CF), and interpersonal skills (IS). It has been externally validated in several subsequent studies [23, 25, 27]. The Chinese version of the PHPQ was developed and validated recently by Yu et al. and has a satisfactory Cronbach’s alpha level of 0.94. [28]. For professional identity, we used the MacLeod Clark Professional Identity Scale to measure how medical students regard themselves as medical professionals. This scale was adapted from the tool originally developed by Brown et al. [29] to measure group identity within a group of factory workers. It has been validated in learners from different health care professions in several studies [28, 30, 31]. For the analysis of teamwork experience, which is a major feature of clinical placement, the Team Understanding Scale (TS) was used. This measurement was first developed by Rentsch et al. to measure students’ understanding of teamwork [32]. It is a ten-item scale and has been shown to correlate significantly with the reported time spent on an individual’s current team. This scale was also validated in other cohorts and shows acceptable reliability [28, 30].

### Statistical analysis

Demographic results are presented as counts (percentages), means (standard deviations, SDs), or medians (interquantile ranges, IQRs), as appropriate. The sums of the measurement item results, such as the total scores or subscale scores, were taken as continuous variables, as suggested by Normal [33]. Comparison of categorical variables between groups was performed using the chi-square test or Fisher’s exact test, as appropriate. Comparison of continuous variables between groups was performed using an independent t test. To better understand the relationship between reported preparedness and other self-reported measurements and the objective learning endpoints collected, the participants were divided into higher and lower groups by the median of the individual relevant indicators, and serial PHPQ results were compared between groups. All statistical analyses were performed using SAS software (version 9.4, SAS Institute Inc., Cary, NC) [34]. A *p* value of less than 0.05 was considered statistically significant.

## Results

**Fig. 1:**
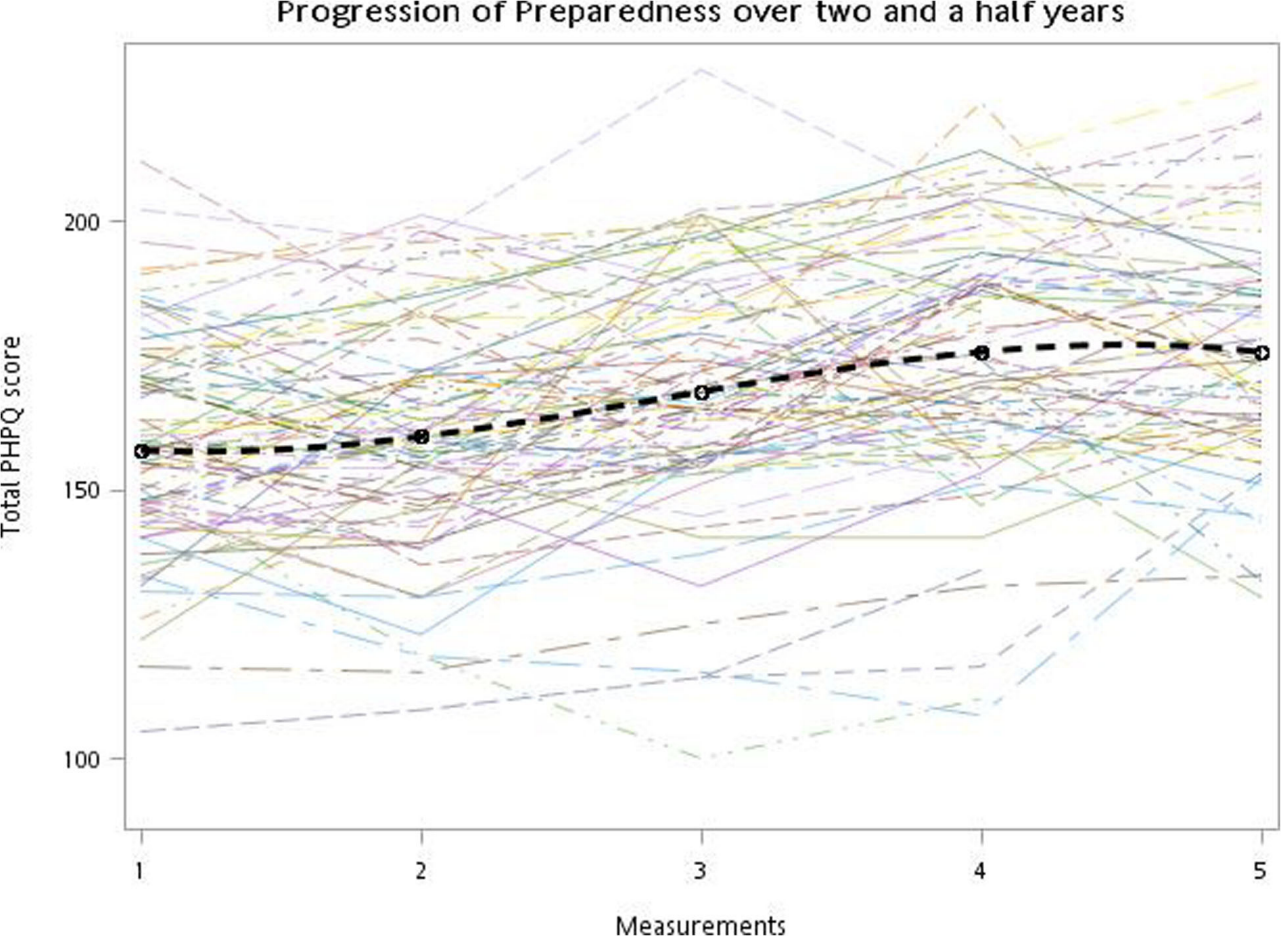
Evolution of the individual total preparedness scores (thin colored lines) and overall average score (black dashed line)

Of the 211 students who graduated in 2019, 85 participated in the study (participation rate 40.3 %). The mean age was 23.6 (SD = 1.25) years, and approximately 60 % of the participants were male. A total of 403 measurements were collected during the study period. The dropout rates of the last two measurements were 3.53 and 20 %, respectively. The descriptive results for the participant characteristics and serial surveys, including the individual domain results, are presented in Table 1. The overall total PHPQ score ranged from 157.3 (SD = 21.2) at the first measurement to 175.5 (SD = 25.6) at the fifth measurement. It is worth noting that the SDs of each measurement increase with time. In Fig. 1, the evolution of the individual total PHPQ scores (thin colored lines) and overall average score (black dashed line) is visualized. As the figure shows, there are both individual differences between learners and temporal differences within the same learner. Despite the variations, an overall gradual increase in the average PHPQ scores was observed from the first to the fourth measurements.

**Table 1:** Descriptive results of the participants and the serial questionnaire measurement results. The numbers are presented as the mean (SD) unless stated otherwise. The questionnaire scales are listed by the scale/subscale name (abbreviation if present, number of items)

	Measurements											
	Overall(*n* = 403)		1st (*n* = 85)		2nd (*n* = 84)		3rd (*n* = 84)		4th (*n* = 82)		5th (*n* = 68)	
age	23.6	(1.25)										
Male^a^	50	(58.8)										
Self-reported academic performance^a^												
Top third	24	(28.2)										
Middle third	41	(48.4)										
Bottom third	20	(23.5)										
Preparedness total (PHPQ, 41)	166.9	(24.1)	157.3	(21.2)	159.9	(21.7)	168.1	(22.3)	175.6	(24.6)	175.5	(25.6)
Interpersonal skills (4)	12.6	(3.53)	11.2	(3.17)	11.7	(3.14)	12.7	(3.16)	13.7	(3.60)	14.0	(3.84)
Confidence (6)	24.1	(4.26)	23.0	(3.82)	23.4	(4.31)	24.0	(4.05)	25.5	(4.22)	24.5	(4.57)
Collaboration (4)	15.7	(3.43)	14.7	(3.35)	14.5	(3.13)	16.0	(2.96)	16.5	(3.52)	17.1	(3.58)
Management (5)	20.2	(4.02)	17.1	(3.12)	17.8	(3.28)	21.0	(3.16)	22.9	(3.20)	22.7	(3.39)
Science (4)	15.1	(2.64)	14.4	(2.42)	14.3	(2.32)	15.3	(2.33)	15.7	(2.62)	16.0	(3.13)
Prevention (6)	27.6	(3.78)	26.8	(3.67)	27.0	(3.70)	27.6	(3.60)	28.2	(3.91)	28.5	(3.83)
Holistic care (6)	25.5	(4.83)	24.3	(5.32)	25.5	(4.60)	25.4	(4.91)	26.1	(4.84)	26.2	(4.17)
Self-directed learning (6)	26.2	(3.94)	25.8	(3.94)	25.7	(3.72)	26.1	(3.94)	27.0	(4.04)	26.6	(4.04)
Professional Identity Scale (PIS, 9)	34.1	(4.02)	33.8	(4.17)	33.7	(3.62)	34.2	(4.17)	34.5	(3.83)	35.0	(4.46)
Team understanding Scale (TS, 10)	34.9	(5.28)	33.0	(5.14)	33.3	(5.50)	35.4	(4.45)	36.5	(4.93)	37.5	(5.10)

^a^presented as count (%)

**Fig. 2:**
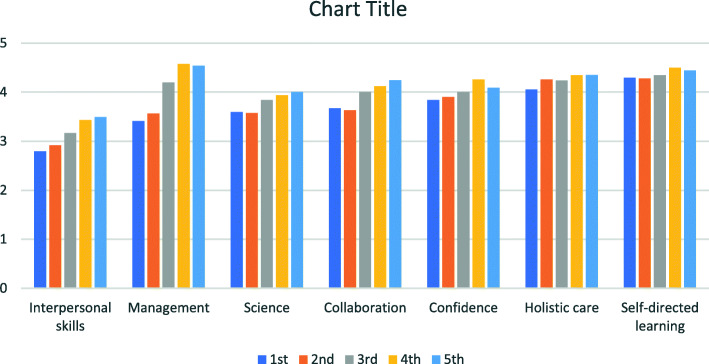
Visualization of the serial average scores of eight PHPQ domains, ordered according to the first measurement score from low to high

In Fig. 2, the serial average scores of the eight domain subscales are shown. The domains of interpersonal skills and patient management are the two in which undergraduate learners generally feel most unprepared in when they first enter clinical rotation. However, the patient management score increased significantly in the subsequent measurements. In contrast, the preparedness for self-directed learning was highest at the first measurement and did not improve much afterwards.

The comparison of the serial PHPQ scores according to the stratification based on self-reported team collaboration and professional identity at the same measurement time is presented in Table 2. From the undergraduate to postgraduate periods, participants with better team collaboration scores showed significantly better preparedness at the first (*p* < 0.01), second (*p* < 0.0001), third (*p* < 0.05), fourth (*p* < 0.0001), and fifth (*p* < 0.01) measurements. Similarly, participants with better professional identity also showed significantly better preparedness at the first (*p* < 0.01), second (*p* < 0.001), third (*p* < 0.05), and fourth (*p* < 0.01) measurements.

**Table 2:** Association of preparedness with other self-reported measurements. Participants were divided into two groups according to the median of the team experience scale and professional identity scale measurements in each measurement, and their preparedness was compared between groups using an independent t test. The average scores are presented as mean (SD)

Measurements	Team understanding scale					Professional Identity Scale				
	Higher	half	Lower	Half	*p*-value	Higher	Half	Lower	half	*p*-value
First	163.0	(20.8)	150.9	(20.0)	0.008^*^	162.9	(22.3)	150.3	(17.7)	0.006^*^
Second	170.0	(18.7)	149.2	(19.5)	< 0.0001^*^	167.4	(16.0)	151.3	(24.2)	0.001^*^
Third	173.8	(22.4)	161.5	(20.5)	0.011^*^	172.8	(17.2)	162.6	(26.1)	0.035^*^
Fourth	184.8	(20.3)	163.6	(24.3)	< 0.0001^*^	181.7	(24.5)	165.2	(21.0)	0.003^*^
Fifth	184.0	(22.3)	166.2	(15.6)	0.006^*^	180.0	(20.2)	173.9	(23.2)	0.353

^*^Statistically significant

To examine the correlation between the objective learning endpoints and the self-reported results, the average learning marks from the last two years of clinical rotation and the national board exam results were used. Participants were divided into two groups by the median scores. The learners with better rotation marks were shown to have better final preparedness (*p* < 0.05) (Table 3). The board exam results, however, were not associated with differences in PHPQ scores. Another self-reported result, previous academic performance, correlated well with the clinical rotation score (*p* < 0.0001) and board exam results (*p* = 0.0001). Sex, on the other hand, has not been shown to affect either the clinical rotation learning marks or the board exam results in the current study.

**Table 3:** Comparison between groups according to objective endpoints. Participants were divided into two groups according to the median of the average clinical rotation learning mark and board exam score. The numbers are presented as count (%) unless stated otherwise

	Learning mark of clinical rotations					Board exam result				
	Higher half		Lower half		*p*-value	Higher half		Lower half		*p*-value
Male	21.0	(50.0)	29	(67.4)	0.1023	16	(55.2)	14	(48.3)	0.5992
Previous performance										
Good	20	(83.3)	4	(16.7)	< 0.0001^*^	15	(79.0)	4	(21.1)	0.0001^*^
Medium	19	(46.3)	22	(53.7)		14	(51.9)	13	(44.2)	
Poor	3	(15.0)	17	(85.0)		0	(0)	12	(100)	
Final PHPQ score^a^	182.7	(29.7)	169.4	(20.1)	0.039^*^	175.9	(20.1)	174.3	(23.5)	0.7833

^a^presented as mean (SD)

^*^statistically significant

## Discussion

The results of this study revealed a gradual overall increasing trend in learners’ preparedness before graduation and the variations among different domains, across individuals, and at different time points. To the best of our knowledge, this is the first quantitative, longitudinal study to measure the change in medical students’ preparedness during the transition from the undergraduate to the postgraduate period. Several similar cohort studies have repeated measurements but mostly focused on either undergraduate or postgraduate periods [35–38]. This study confirms the relationship of preparedness for practice with other subjective indicators, such as the perception of professional identity and team collaboration experience. This study also demonstrated the association of subjective endpoints, such as survey results and self-reported previous academic performance, with objective learning outcomes, namely, clinical rotation marks.

Preparedness concerns all aspects of ability and is thought to be linked to increased feelings of self-efficacy [19, 20]. The current study confirmed the association between preparedness and other subjective indicators. In other words, the more prepared students felt, the greater they perceived themselves as doctors and the greater they were integrated into the medical team. The above association creates a virtuous cycle, which may ultimately affect the learner’s relationship with his or her supervisor and medical team members. This also explains why preparedness is associated with one of the objective learning endpoints, the average clinical rotation mark. A certain proportion of the composition of the rotation mark consists of subjective evaluations from the supervisor. As Chipchase et al. mentioned, supervision of a poorly prepared student is demanding, and a successful relationship during clinical learning also falls to students, who must present themselves as competent, professional, and well prepared [12, 39].

Traditionally, preparedness is assessed and licenced through medical board examinations, which are focused primarily on the mastery of requisite medical knowledge [2, 3, 25]. Other critical domains and skills, such as team collaboration, communication, technical skills, and the ability to provide holistic care, are not assessed to the same degree. The board exam MCQ test scores were not associated with differences in preparedness in the current study, indicating that real-life patient care, not medical knowledge, is often the main domain in which students feel unprepared. This was also evidenced in the current study by the fact that the subscale of patient management showed the sharpest increase among all eight domains. Likewise, previous studies have indicated that medical graduates often feel unprepared for common clinical procedures due to insufficient “hands-on” practice [14, 20, 28]. Fortunately, in recent decades, newer clinically oriented assessments, such as the OSCE (Objective Structured Clinical Examination), DOPS (Direct Observation of Procedural Skills), and Mini-CEX (Mini Clinical Evaluation Exercise), have been added to medical curriculums as formative or summative assessments. In addition, many countries have adapted skill assessments as part of the board licencing process [1, 2, 5]. These efforts may help clinical teachers focus their training on cultivating a competent young doctor as a whole rather than only on increasing medical knowledge.

The current study showed a steady overall upward trend in medical students’ preparedness as they spent more time in clinical rotation and gained more experience. However, these developments were not necessarily linear or unidirectional. Similar findings were reported by Monrouxe et al. in their qualitative research: challenging circumstances sometimes made undergraduates feel unprepared, even for situations where they had previously indicated preparedness [40]. A number of factors related to training programs and clinical workplace environments were identified previously as being able to facilitate students’ perceptions of feeling prepared for practice. These include close identification with role models, opportunities for shadowing seniors and relevant workplace teaching and support [9, 23, 41]. Clinical teachers must understand that there are ups and downs in the process of learning and transition, just as there are prosperity and adversity in real life.

Our results also showed that the overall PHPQ scores of 1 month before graduation and five months after graduation were approximately the same. This result is somewhat different from a recent article by Burridge et al. in which UK postgraduate doctors reported their first experience of genuine preparedness between three and six months [42]. A possible explanation could be that there is a two-month gap between graduation and PGY training in Taiwan. Another possibility could be due to individual differences with extreme values, evidenced by the increase in SDs of the overall PHPQ scores. At the systemic level, efforts should be made to create a supportive learning environment. Individuals with significantly lower preparedness should be identified, as this characteristic was reported to be associated with emotional exhaustion and psychological distress [43].

### Limitations

This study has several limitations. First, we recruited volunteer medical students, and selection bias may therefore exist, as the participants may not represent the whole population in terms of their knowledge, skills, and preparedness for practice. Second, we utilized mainly self-report surveys, and some of the results may not reflect the actual situation of the participants. Nevertheless, the authors tried their best to link and analyze the subjective results with objective learning endpoints. Third, this study is a single-nation study, and contextual differences must be taken into consideration before applying the results of this study. Further longitudinal studies in different contexts may be beneficial to understanding the relationship between cultural differences and preparedness for clinical practice among medical students.

## Conclusions

The preparedness for practice of medical students from the undergraduate to postgraduate periods is associated with their professional identity, teamwork experience, and objective clinical rotation endpoints. Although preparedness generally increases with time, educators must understand that there are temporal fluctuations and individual differences in learners’ preparedness.

## Data Availability

The datasets used and analyzed during the current study are available from the corresponding author on a reasonable request.

## Abbreviations

PGY: Postgraduate year
MCQ: Multiple choice questions
OSCE: Objective Structured Clinical Examination
DOPS: Direct Observation of Procedural Skills
Mini-CEX: Mini Clinical Evaluation Exercise

## References

[1] DeZee KJ, Artino AR, Elnicki DM, Hemmer PA, Durning SJ (2012). Medical education in the United States of America. Med Teach.

[2] Chou J-Y, Chiu C-H, Lai E, Tsai D, Tzeng C-R (2012). Medical education in Taiwan. Med Teach.

[3] Kozu T (2006). Medical education in Japan. Acad Med.

[4] Ten Cate O (2007). Medical education in the Netherlands. Med Teach.

[5] Nikendei C, Weyrich P, Jünger J, Schrauth M (2009). Medical education in Germany. Med Teach.

[6] Moczko TR, Bugaj TJ, Herzog W, Nikendei C (2016). Perceived stress at transition to workplace: a qualitative interview study exploring final-year medical students’ needs. Adv Med Educ Pract.

[7] Blackwell B (1986). Prevention of impairment among residents in training. JAMA.

[8] Monrouxe LV, Grundy L, Mann M, John Z, Panagoulas E, Bullock A (2017). How prepared are UK medical graduates for practice? A rapid review of the literature 2009–2014. BMJ Open.

[9] Brennan N, Corrigan O, Allard J, Archer J, Barnes R, Bleakley A (2010). The transition from medical student to junior doctor: today’s experiences of Tomorrow’s Doctors. Medical education.

[10] Ernstzen D, Bitzer E, Grimmer-Somers K (2009). Physiotherapy students’ and clinical teachers’ perceptions of clinical learning opportunities: A case study. Med Teach.

[11] Rodger S, Webb G, Devitt L, Gilbert J, Wrightson P, McMeeken J (2008). Clinical education and practice placements in the allied health professions: an international perspective. J Allied Health.

[12] Chipchase LS, Buttrum PJ, Dunwoodie R, Hill AE, Mandrusiak A, Moran M (2012). Characteristics of student preparedness for clinical learning: clinical educator perspectives using the Delphi approach. BMC Med Educ.

[13] Jones OM, Okeke C, Bullock A, Wells SE, Monrouxe LV. ‘He’s going to be a doctor in August’: a narrative interview study of medical students’ and their educators’ experiences of aligned and misaligned assistantships. BMJ Open. 2016;6(6):e011817. 10.1136/bmjopen-2016-011817 PMC4908916 27288387

[14] Goldacre MJ, Taylor K, Lambert TW (2010). Views of junior doctors about whether their medical school prepared them well for work: questionnaire surveys. BMC Med Educ.

[15] Lundin RM, Bashir K, Bullock A, Kostov CE, Mattick KL, Rees CE, et al. “I’d been like freaking out the whole night”: exploring emotion regulation based on junior doctors’ narratives. Adv Health Sci Educ Theory Pract. 2017;23(1):7–28. 10.1007/s10459-017-9769-y PMC5801373 28315113

[16] Phillips DP, Barker GE (2010). A July spike in fatal medication errors: a possible effect of new medical residents. J Gen Intern Med.

[17] Young JQ, Ranji SR, Wachter RM, Lee CM, Niehaus B, Auerbach AD. “July effect”: impact of the academic year-end changeover on patient outcomes: a systematic review. Ann Intern Med. 2011;155(5):309–15. 10.7326/0003-4819-155-5-201109060-00354 21747093

[18] Burford B, Whittle V, Vance GH (2014). The relationship between medical student learning opportunities and preparedness for practice: a questionnaire study. BMC Med Educ.

[19] Murdoch-Eaton D, Whittle S (2012). Generic skills in medical education: developing the tools for successful lifelong learning. Medical education.

[20] Cantor JC, Baker LC, Hughes RG (1993). Preparedness for practice: young physicians’ views of their professional education. Jama.

[21] Hill J, Rolfe IE, Pearson SA, Heathcote A (1998). Do junior doctors feel they are prepared for hospital practice? A study of graduates from traditional and non-traditional medical schools. Medical education.

[22] Illing J, Morrow G, Kergon C, Burford B, Spencer J, Peile E, et al. How prepared are medical graduates to begin practice. A comparison of three diverse UK medical schools. Final report to GMC April 2008. 2008.

[23] Dean SJ, Barratt AL, Hendry GD, Lyon PM (2003). Preparedness for hospital practice among graduates of a problem-based, graduate-entry medical program. Med J Aust.

[24] Morrow G, Johnson N, Burford B, Rothwell C, Spencer J, Peile E (2012). Preparedness for practice: the perceptions of medical graduates and clinical teams. Med Teach.

[25] Bojanic K, Schears GJ, Schroeder DR, Jenkins SM, Warner DO, Sprung J (2009). Survey of self-assessed preparedness for clinical practice in one Croatian medical school. BMC Res Notes.

[26] Hill J, Rolfe IE, Pearson SA, Heathcote A (1998). Do junior doctors feel they are prepared for hospital practice? A study of graduates from traditional and non-traditional medical schools. Med Educ.

[27] MacCarrick G, Kelly C, Conroy R (2010). Preparing for an institutional self review using the WFME standards–An International Medical School case study. Med Teach.

[28] Yu SR, Cheng YC, Tseng HM, Chang YC, Ma SD, Huang CD (2020). Undergraduates’ preparedness for practice is associated with professional identity and perception of educational environment: A validation study. Biomed J.

[29] Brown R, Condor S, Mathews A, Wade G, Williams J (1986). Explaining intergroup differentiation in an industrial organization. J Occup Psychol.

[30] Adams K, Hean S, Sturgis P, Clark JM (2006). Investigating the factors influencing professional identity of first-year health and social care students. Learn Health Soc Care.

[31] Worthington M, Salamonson Y, Weaver R, Cleary M (2013). Predictive validity of the Macleod Clark Professional Identity Scale for undergraduate nursing students. Nurse Educ today.

[32] Rentsch J (1993). editor Predicting team effectiveness from teamwork schema similarity.

[33] Norman G (2010). Likert scales, levels of measurement and the “laws” of statistics. Advances in health sciences education.

[34] SAS Institude Inc., SAS 9.4 Documentation 2017 [Available from: http://support.sas.com.

[35] Gouda P, Kitt K, Evans DS, Goggin D, McGrath D, Last J, et al. Irish medical students’understanding of the intern year. Ir Med J. 2016;109(4):387–9. 27685481

[36] Laven G, Keefe D, Duggan P, Tonkin A (2014). How was the intern year?: self and clinical assessment of four cohorts, from two medical curricula. BMC Med Educ.

[37] Dare A, Fancourt N, Robinson E, Wilkinson T, Bagg W (2009). Training the intern: the value of a pre-intern year in preparing students for practice. Med Teach.

[38] Kelly C, Noonan CL, Monagle JP (2011). Preparedness for internship: a survey of new interns in a large Victorian health service. Aust Health Rev.

[39] Cross V (1998). Begging to differ? Clinicians’ and academics’ views on desirable attributes for physiotherapy students on clinical placement. Assess Eval Higher Educ.

[40] Monrouxe L, Bullock A, Cole J, Gormley G, Kaufhold K, Kelly N, et al. How Prepared are UK Medical Graduates for Practice? Final report from a programme of research commissioned by the General Medical Council. General Medical Council; 2014.

[41] Cave J, Woolf K, Jones A, Dacre J (2009). Easing the transition from student to doctor: how can medical schools help prepare their graduates for starting work?. Med Teach.

[42] Burridge S, Shanmugalingam T, Nawrozzadeh F, Leedham-Green K, Sharif A (2020). A qualitative analysis of junior doctors’ journeys to preparedness in acute care. BMC Med Educ.

[43] Willcock SM, Daly MG, Tennant CC, Allard BJ (2004). Burnout and psychiatric morbidity in new medical graduates. Med J Aust.

